# Identification of multiple salicylic acid-binding proteins using two high throughput screens

**DOI:** 10.3389/fpls.2014.00777

**Published:** 2015-01-12

**Authors:** Murli Manohar, Miaoying Tian, Magali Moreau, Sang-Wook Park, Hyong Woo Choi, Zhangjun Fei, Giulia Friso, Muhammed Asif, Patricia Manosalva, Caroline C. von Dahl, Kai Shi, Shisong Ma, Savithramma P. Dinesh-Kumar, Inish O'Doherty, Frank C. Schroeder, Klass J. van Wijk, Daniel F. Klessig

**Affiliations:** ^1^Boyce Thompson Institute for Plant Research, Cornell UniversityIthaca, NY, USA; ^2^Plant, Soil, and Nutrition Laboratory, United States Department of AgricultureIthaca, NY, USA; ^3^Department of Plant Biology, Cornell UniversityIthaca, NY, USA; ^4^Department of Plant Biology and Genome Center, University of California, DavisDavis, CA, USA

**Keywords:** salicylic acid, salicylic acid-binding proteins, salicylic acid signaling, plant immunity, disease resistance

## Abstract

Salicylic acid (SA) is an important hormone involved in many diverse plant processes, including floral induction, stomatal closure, seed germination, adventitious root initiation, and thermogenesis. It also plays critical functions during responses to abiotic and biotic stresses. The role(s) of SA in signaling disease resistance is by far the best studied process, although it is still only partially understood. To obtain insights into how SA carries out its varied functions, particularly in activating disease resistance, two new high throughput screens were developed to identify novel SA-binding proteins (SABPs). The first utilized crosslinking of the photo-reactive SA analog 4-AzidoSA (4AzSA) to proteins in an Arabidopsis leaf extract, followed by immuno-selection with anti-SA antibodies and then mass spectroscopy-based identification. The second utilized photo-affinity crosslinking of 4AzSA to proteins on a protein microarray (PMA) followed by detection with anti-SA antibodies. To determine whether the candidate SABPs (cSABPs) obtained from these screens were true SABPs, recombinantly-produced proteins were generated and tested for SA-inhibitable crosslinking to 4AzSA, which was monitored by immuno-blot analysis, SA-inhibitable binding of the SA derivative 3-aminoethylSA (3AESA), which was detected by a surface plasmon resonance (SPR) assay, or SA-inhibitable binding of [^3^H]SA, which was detected by size exclusion chromatography. Based on our criteria that true SABPs must exhibit SA-binding activity in at least two of these assays, nine new SABPs are identified here; nine others were previously reported. Approximately 80 cSABPs await further assessment. In addition, the conflicting reports on whether NPR1 is an SABP were addressed by showing that it bound SA in all three of the above assays.

## Introduction

Salicylic acid (SA) and its derivatives, collectively termed salicylates, have been the focus of the medical community since the discovery in 1828/1829 by German and French pharmacologists that the active ingredient in willow bark, which relieves pain and fever, is salicin, a glycoside of SA. Salicin is rapidly converted into SA in the gastrointestinal tract. Over the next decade, German and French chemists produced synthetic SA, which greatly reduced SA's cost and widened its use. The synthesis of acetylSA (aspirin), which causes less stomach irritation than SA but is comparably efficacious, enabled this compound to become the most widely used drug worldwide (natural or synthetic; reviewed in Weissmann, 1991; Wick, 2012).

Efforts to characterize SA's role in plants span a much more recent history. For centuries, SA and other phenolic compounds synthesized by plants were thought to be non-essential for critical processes and thus called secondary metabolites (Hadacek et al., 2011). Three discoveries changed this view. First, Cleland and Ajami (1974) identified SA in the phloem sap of flowering *Xanthium strumarium*. Since this sap induced flowering in *Lemna gibba*, they suggested that SA is an endogenous signal. Second, Raskin and colleagues (reviewed in Raskin, 1992) demonstrated that a dramatic rise in SA levels preceded thermogenesis in the central column of the inflorescence of *Sauromatum guttatum*. Moreover, application of exogenous SA caused elevated temperatures in this organ, suggesting that SA is an important signaling molecule for thermogenesis in some plants. Third, analyses of disease resistance in the tobacco-tobacco mosaic virus pathosystem (White, 1979; Malamy et al., 1990) and the cucumber-tobacco necrosis virus/*Colletotrichum lagenarium* pathosystems (Meìtraux et al., 1990), followed by many studies over the following two decades (reviewed in Vlot et al., 2009), demonstrated that SA is a critical signaling hormone for the activation of several levels of immunity in response to biotrophic pathogens, including effector-trigger immunity (also called *R* gene-mediated resistance), Microbe-Associated Molecular Pattern (MAMP)-triggered immunity, and systemic acquired resistance. Thousands of papers documenting SA's involvement in plant disease resistance have been published over the past half century; this extensive research has revealed a complex signaling network of upstream and downstream components (reviewed in Vlot et al., 2009; Dempsey et al., 2011). In addition to its many roles in immunity and its involvement in thermogenesis and flowering, SA has been shown to play an important role(s) in responding to abiotic stresses, such as heat, chilling, drought, osmotic stress, and heavy metal toxicity. SA also regulates biochemical and physiological processes throughout a plant's life span, including seed germination, photosynthesis, respiration, growth, and senescence (reviewed in Rivas-San Vicente and Plasencia, 2011).

Several general approaches have been used to decipher how SA modulates the plant immune system. The first involved the isolation of mutants, primarily in Arabidopsis, that exhibited altered defenses-related responses following exogenous SA treatment. The most notable success of this genetic approach was the identification of NPR1/NIM1/SAI1 by four independent research groups (Cao et al., 1994; Delaney et al., 1995; Glazebrook et al., 1996; Shah et al., 1997). The pioneering work of Dong and co-workers demonstrated that NPR1 is a transcriptional co-factor that plays a critical role in positively regulating SA-induced immune responses (for review Spoel and Dong, 2012). The second approach utilized classical biochemical methods to identify proteins that bound radio-labeled SA in protein extracts prepared primarily from tobacco leaves. This approach yielded several SA-binding proteins (SABPs), all of which are enzymes. They include catalase and ascorbate peroxidase, which are the two major H_2_O_2_-scavenging enzymes, as well as carbonic anhydrase (named SABP3), and methyl salicylate esterase (named SABP2), which is involved in systemic acquired resistance (Chen et al., 1993; Durner and Klessig, 1995; Slaymaker et al., 2002; Kumar and Klessig, 2003; Park et al., 2007). The third approach used genetic and biochemical methods to assess whether SA directly/physically interacts with NPR1 and/or its paralogs NPR3 and NPR4. Fu et al. (2012) reported that while NPR1 did not bind SA, NPR3 and NPR4 did, and therefore concluded that NPR3 and NPR4 are receptors for SA. In contrast, Wu et al. (2012) demonstrated that NPR1 bound SA and thus concluded that it is an SA receptor.

While these efforts to identify SA receptors have provided important insights into SA's mechanisms of action during immune responses, many aspects of SA signaling remain unclear. Beyond determining whether NPR1, NPR3 and/or NPR4 function as SA receptors, some SA-induced defense responses are activated via an NPR1-independent pathway that is currently uncharacterized. Likewise, the mechanisms through which SA modulates many other NPR1-independent plant processes are unknown. To facilitate the identification of proteins through which SA mediates its effects on these processes, we developed two high-throughput strategies to identify putative/candidate SABPs (cSABPs) in Arabidopsis using biochemical and biophysical methods. The first relies on photo-affinity crosslinking to 4-Azido SA (4AzSA), followed by immuno-selection and mass spectroscopy-based identification (Tian et al., 2012), while the second utilizes 4AzSA crosslinking and immuno-detection of cSABPs on a protein microarray (PMA) (Moreau et al., 2013). Here, we report the identification of nine new SABPs, based on at least two independent assays, and provide a list of more than 100 cSABPs identified by these two high-throughput screens.

## Method and materials

### Plant growth and pathogen inoculation

*Arabidopsis thaliana* plants were grown as described previously (Vlot et al., 2008). Pathogen inoculation and leaf harvest were performed as described previously (Tian et al., 2012).

### Plasmid construction, protein expression and purification

cSABPs were selected for further analyses in part based on the absence of predicted trans-membrane domains by TMHMM (http://www.cbs.dtu.dk/services/TMHMM/). PCR amplified protein coding sequences from selected cSABPs were cloned into pET28a to generate recombinant proteins with an N-terminal His_6_ tag. To increase the solubility of NPR1 and FBA5, N-terminal fusions to His_6_-MBP (maltose binding protein) were generated in the pET-MALHT vector. The error-free clones were confirmed by sequencing and then transformed into either BL21 (DE3) or Rosetta2 (DE3) (Novagen) *E. coli* strains for protein expression. The bacteria were grown at 37°C in 2 liters of LB containing 50 μg/ml kanamycin for BL21 (DE3) or 50 μg/ml kanamycin and 34 μg/ml chloramphenicol for Rosetta 2(DE3) cells to an OD_600_ of 0.6, before addition of IPTG to a final concentration of 0.1–1 mM to induce gene expression. Induced cultures were incubated overnight at 20° C. The cells were then harvested by centrifugation and the pellet was resuspended into the lysis buffer (50 mM tris pH 7.4, 500 mM NaCl, 10% glycerol, 20 mM Imidazole, 0.5% triton X-100 and 1 mM PMSF). Resuspended cells were disrupted by sonication and cell debris was removed by centrifugation. The clarified supernatant was incubated with Ni-NTA His resin (Novagen) for 1 h, then washed with approximately 40 bed volumes of lysis buffer containing increasing concentrations (20, 30, and 40 mM) of imidazole. The remaining proteins bound to the Ni-NTA resin were eluted in lysis buffer supplemented with 250 mM of imidazole. Eluted proteins were concentrated and subjected to gel filtration using a HiLoad 16/600 superdex 200 prep grade column (GE healthcare). Fractions containing the purified protein were collected, pooled, aliquoted, and stored at −80° C until use.

### Isolation and identification of 4AzSA-crosslinked proteins by immuno-selection and mass spectroscopy

4AzSA-crosslinked proteins were isolated and identified as described previously (Tian et al., 2012).

### Identification of SA-binding proteins via SA affinity chromatography

SA-immobilized resin was prepared using a PharmaLink Immobilization Kit (Pierce), according to the manufacturer's instructions. The coupling with 0.5–1 mg SA typically resulted in ~180 μg SA immobilized per mL resin. Protein extract from Arabidopsis leaves were suspended in loading buffer (50 mM KPO_4_ (pH 8.0) containing 50 mM NaCl, a protease inhibitor cocktail (Sigma) and 0.1 % (v/v) Triton X-100) and loaded onto a column containing the SA-linked resin. The loaded column was washed with loading buffer without and then with 0.1–10 mM 4-HBA to remove non-specifically bound proteins. Column-retained proteins were eluted with loading buffer containing 5 mM SA, and analyzed by SDS-PAGE. Eluted proteins were identified by mass spectroscopy.

### Identification of 4AzSA-crosslinked proteins by PMA

*Arabidopsis* TAP-tagged recombinant purified proteins were printed to produce high density Arabidopsis microarrays (Popescu et al., 2007). For identification of cSABPs, the Arabidopsis PMA chips, each containing 10,000 proteins printed in duplicate, were blocked using protein-free blocking buffer (PFBB; Thermo Fisher Scientific) for 1 h at 4° C. After applying PFBB containing or lacking 500 μM 4AzSA, the PMAs were incubated in the dark for 30 min at room temperature before irradiation with 250 mJ UV light using a GS GENE linker™ UV chamber (Bio-Rad). Irradiated PMAs were washed twice for 5 min with PFBB, twice for 5 min with PBS plus 0.1% Tween 20, and twice for 5 min with PFBB. The PMAs were then incubated at 4° C overnight with sheep α-SA antibody (1:2000 in PFBB; AbD Serotec) without shaking. For washing the PMAs were incubated twice for 5 min with PFBB, twice for 5 min with PBS plus 0.1% Tween 20, and twice for 5 min with PFBB. PMAs were then incubated with Dylight 649 conjugated donkey α-sheep secondary antibody (1:5000 in PFBB; Jackson ImmunoResearch) at RT for 1 h followed by six 5-min washing steps using PBS plus 0.1% Tween 20 and two 5-min steps with distilled water. Dried PMAs were scanned using a GenePix4000B scanner (Molecular Devices), and the data were collected using GenePix Pro 6.0 software (Molecular Devices).

### Statistical analysis for the identification of 4AzSA-crosslinked proteins by PMA

Microarray data were normalized using the quantile normalization method (Bolstad et al., 2003). Differential binding to the α-SA antibody of proteins with or without 4AzSAcrosslinking was determined with LIMMA (Smyth, 2004). Raw *p*-values of multiple tests were corrected using false discovery rate (FDR) (Benjamini and Hochberg, 1995). Proteins with FDR < 0.01 were identified as cSABPs.

### Assessment of 3AESA-binding activity by SPR

SPR analyses of 3AESA binding and competition by SA were performed with a Biacore 3000 instrument (GE Healthcare). Immobilization of 3AESA on the CM5 sensor chip was performed as described previously (Tian et al., 2012). Activation, deactivation, and preparation of the mock coupled flow cell were performed by using amine coupling kit using manufacturer guidelines (GE healthcare). Briefly, carboxyl group of CM5 sensor chip was activated by using a mixture of 1-ethyl-3-(3-dimethyl aminopropyl) carbodiimide hydrochloride (EDC) and N—hydroxy-succinimide (NHS) for the period of 7 min at a flow rate of 5 μl/min. After activation of sensor chip, 10 mM of 3-AESA dissolved in 0.1 M borate buffer, pH 10 was passed over for the period of 30 min at a flow rate of 5 μl/min for immobilization.Next excess reactive groups was inactivated by flowing ethanolamine hydrochloride-NaOH pH 8.5 for the period of 7 min. at a flow rate of 5 μl/min. HBS-EP buffer (0.01 M HEPES, ph 7.4. 0.15 M NaCl, 3 mM EDTA, 0.005% surfactant P20; GE healthcare) was used as a running buffer in all assays. To test SA binding of cSABPs, proteins were filtered and diluted in HBS-EP buffer with or without various concentrations of SA, and then flowed through the flow cell of sensor chip with 3-AESA immobilized or through the mock-coupled flow cell. The binding signal was generated by subtracting the signal generated by mock-coupled flow cells from that generated with the 3-AESA immobilized flow cell. The flow cells were regenerated by stripping off bound protein by flowing NaOH solution (pH12).

### Assessment of SA-binding activity by photo-affinity labeling

SA-binding activity was assessed by photo-affinity labeling as described previously (Tian et al., 2012). Briefly, purified proteins (2 μg) were incubated 1 h on ice with 4AzSA (50 μM) in 40 μl 1X PBS without or with various concentrations of excess SA, followed by UV irradiation with 254 nm UV light at an energy level of 30 mJ using a GS GENE linker™ UV chamber (Bio-Rad). 10 μl of reaction mixture were subjected to SDS-PAGE followed by immuno-blotting with α-SA antibody (Novus Biologicals) to detect 4AzSA-crosslinked proteins.

### [^3^H]SA-binding assays and determination of binding affinity between NPR1 and SA

[^3^H]SA-binding assays were performed using size exclusion chromatography as described previously (Chen and Klessig, 1991). Briefly, pre-equilibrate sephadex™ G-25 (GE healthcare) with PBS buffer containing 0.1% Tween-20 overnight at 4° C. Size-exclusion column was prepared using 1 ml syringe with glass wool fiber as filter and packed with overnight equilibrated sephadex™ G-25; excess buffer was removed by centrifugation. Binding of [^3^H]SA with His_6_-MBP-tagged NPR1, His_6_-MBP-tagged FER1, MBP and no protein control was performed in PBS buffer with 100 μl reaction volume in the absence or presence of excess unlabeled SA (10,000-fold). The reaction mix was incubated on ice for 1 h, and then loaded on the column and centrifuged. The flow through was collected and dissolved in scintillation liquid and radioactivity was measured by a scintillation counter (LS6500; Beckman Coulter, Pasadena, CA). The Kd value was determined by non-linear fitting model of Michaelis-Menten equation with [^3^H]SA concentration from 5 to 640 nM using OriginPro 7.5 software (OriginLab, Northampton, MA).

## Results

### Identification of cSABPs by immuno-selection of 4AzSA crosslinked proteins and affinity chromatography with an SA-linked matrix

Over the past several years, we have conducted four large-scale screens using a previously described strategy with 4AzSA (Tian et al., 2012), and three large-scale screens using SA linked to a matrix for affinity chromatography. It should be noted that 4AzSA is a biologically active SA analog which mimics SA function in plants and is bound by previously identified SABP such as MES9 (Tian et al., 2012). For the 4AzSA screen, soluble protein extracts prepared from Arabidopsis leaves were incubated with 4AzSA; UV irradiation was then used to covalently crosslink 4AzSA to the proteins binding it. The 4AzSA-crosslinked proteins were selected with antibodies directed against SA, and the selected proteins were identified by mass spectroscopy. The SA affinity chromatography selection was performed by loading soluble protein extracts prepared from Arabidopsis leaves onto a column containing SA immobilized on a matrix. After washing the column with the biologically inactive SA analog 4-hydroxy benzoic acid to remove non-specifically bound proteins, the remaining proteins bound to the SA matrix were eluted with 5 mM SA and identified by mass spectroscopy. Through these seven screens, 35 proteins were identified two or more times, including at least once via crosslinking to 4AzSA (Table 1). The proteins represent 26 different protein families, and include catalase and carbonic anhydrase, which were previously identified as SABPs in tobacco (Chen et al., 1993; Slaymaker et al., 2002). To determine whether these proteins represent true SABPs, the encoding genes for 19 SABPs were obtained and successfully expressed in *E. coli*. The His_6_-tagged recombinant proteins, which were purified by affinity chromatography on a Ni matrix followed by size fractionation on a 16/600 superdex 200 column, were then tested for SA-binding activity, primarily by assessing SA-inhibitable binding to 4AzSA, which was detected with anti-SA antibodies using immuno-blotting, and by monitoring SA-inhibitable binding to a 3AESA-bound sensor chip, which was detected by SPR.

**Table 1:**
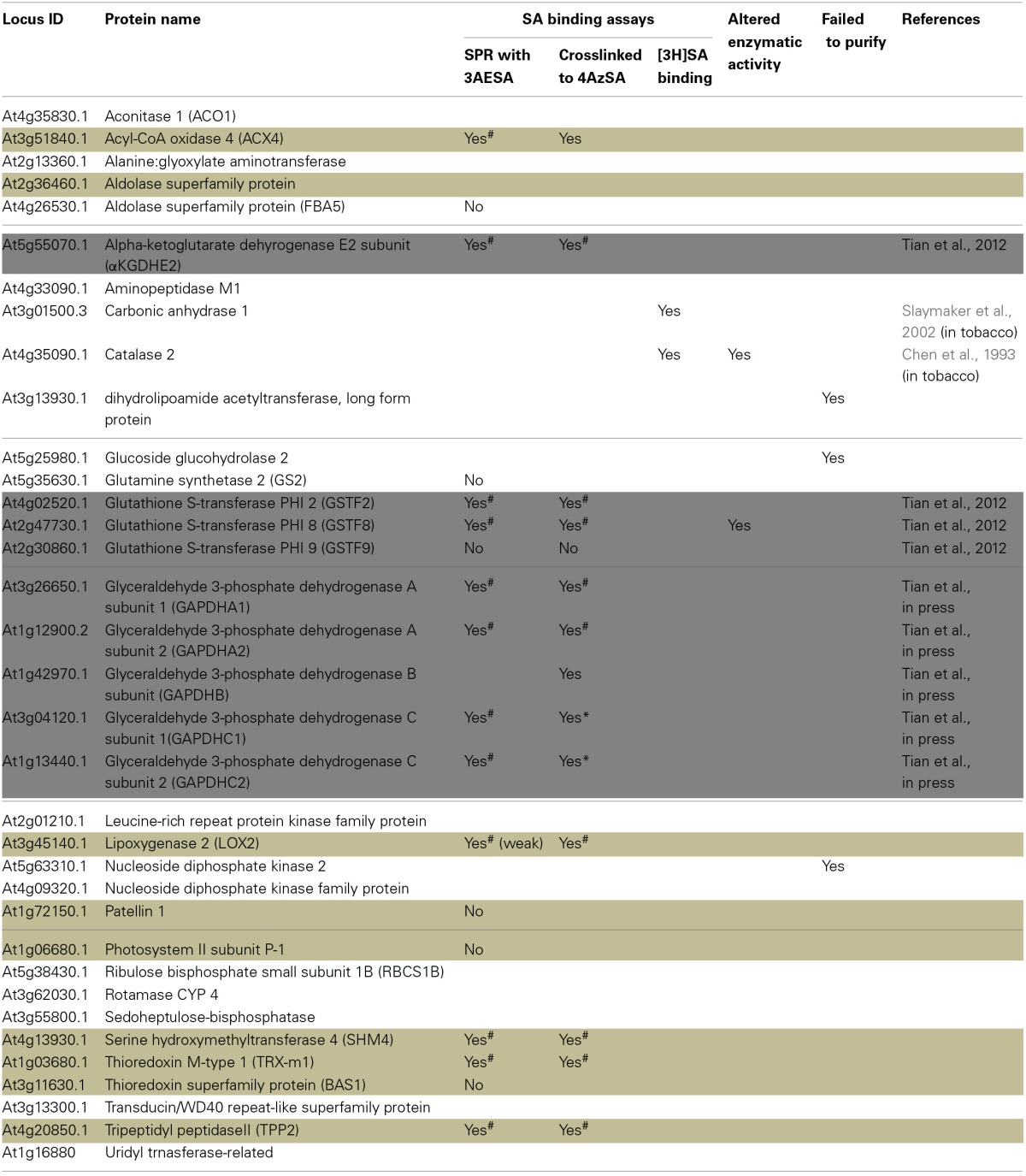
**List of cSABPs identified by photo-activated crosslinking to 4AzSA and immuno-selection**.

Proteins listed here were identified two or more times in seven large scale screens involving crosslinking to 4AzSA followed by immune-selection or using affinity chromatography with an SA-linked matrix.

^#^Competable with SA.

^*^Enhanced by SA.

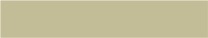
Chracterized in this study.

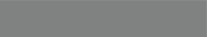
Previousaly reported cSABPs/SABPs.

**Figure 1:**
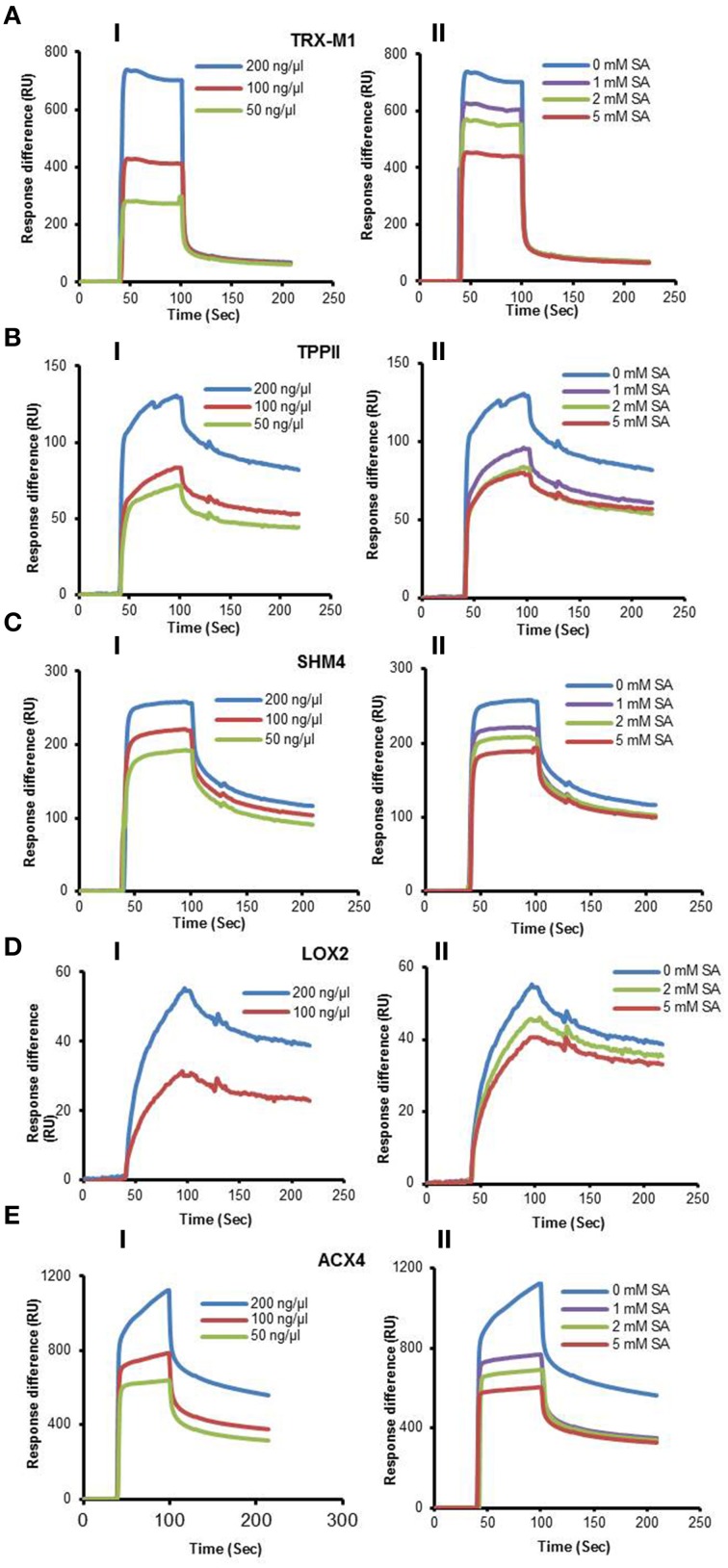
**SPR analyses of cSABPs identified by immuno-selection screens of 4AzSA crosslinked proteins**. (i) Sensorgrams obtained with three concentrations (50, 100 or 200 ng/μl) of each recombinant, purified cSABP on a 3AESA-immobilized sensor chip for **(A)** TRX-m1, **(B)** TPPII, (**C**) SHM4, **(D)** LOX2, and **(E)** ACX4. (ii) Sensorgrams for each cSABP (200 ng/μl) in the absence (0 mM) or presence of three concentrations of SA (1, 2 or 5 mM) using a 3AESA-immobilized chip. The signals detected from a mock-coupled control chip were subtracted.

**Figure 2:**
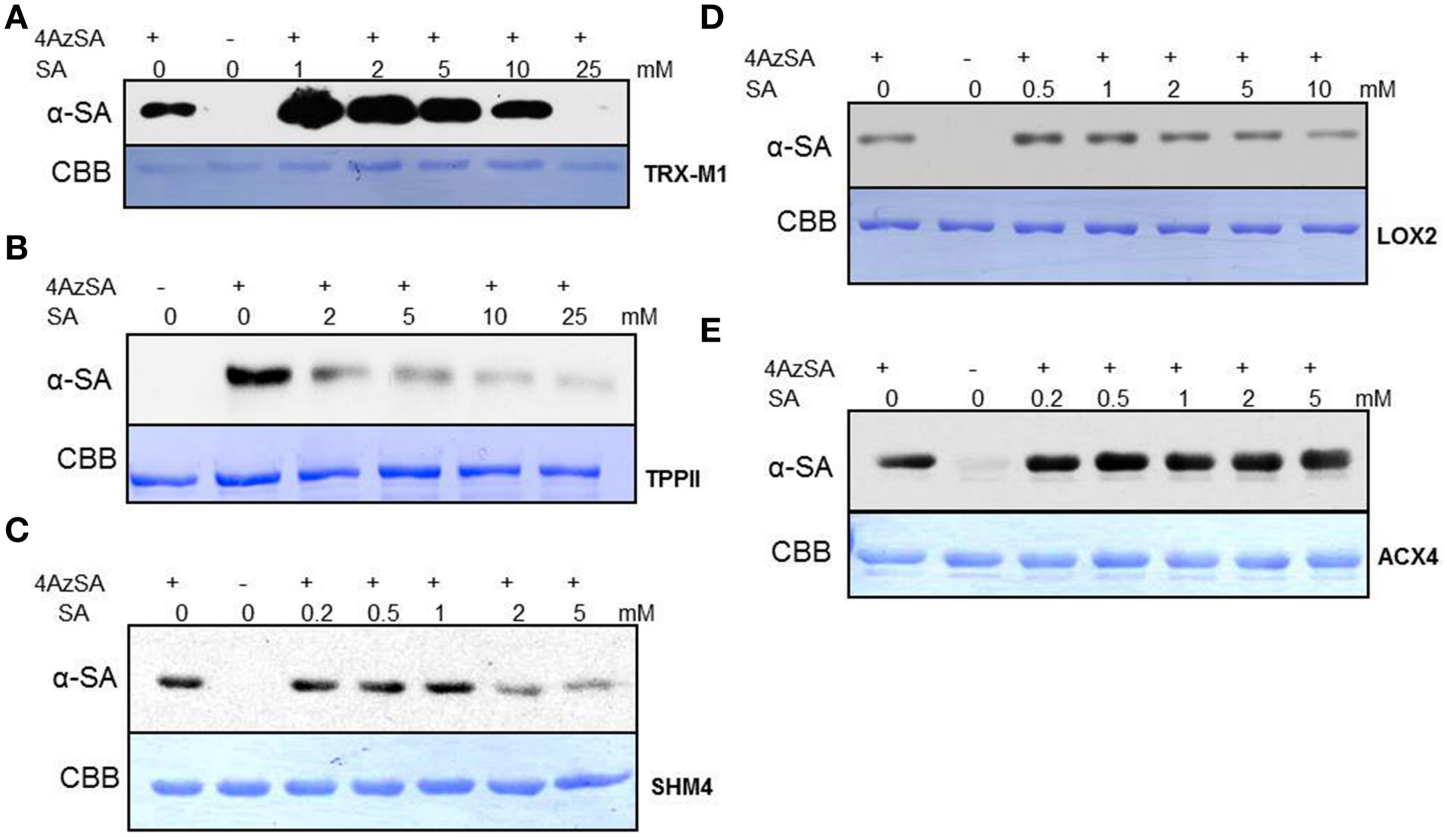
**Immuno-blot analyses monitoring SA competition of 4AzSA crosslinking to recombinant cSABPs identified by the 4AzSA/immuno-selection screen**. Photo-activated crosslinking of 50 ng/μl of the indicated recombinant purified proteins: **(A)** TRX-m1, **(B)** TPPII, **(C)** SHM4, **(D)** LOX2, and **(E)** ACX4 to 4AzSA (50 μM) in the absence or presence of increasing amounts of SA was detected by immuno-blotting using an α-SA antibody. Reactions without 4AzSA served as controls. Note that controls with 4AzSA but without photo-activation were previously shown to give similar results (Tian et al., 2012). Proteins stained with Coomassie brilliant blue (CBB) served as the loading control.

Here we report the analysis of 10 cSABPs: acyl-CoA oxidase 4 (ACX4), aldolase superfamily protein (FBA5), glutamine synthetase 2 (GS2), lipoxygenase 2 (LOX2), patellin 1, photosystem II subunit P-1, serine hydroxymethyltransferase 4 (SHM4), thioredoxin-m1 (TRX-m1), thioredoxin superfamily protein BAS1, and tripeptidyl peptidase II (TPPII). The results for nine more cSABPs, including α-ketoglutarate dehydrogenase, the glutathione S-transferase PHI family (Tian et al., 2012) and glyceraldehyde 3-phosphate dehydrogenase family, are included in Table 1 but their characterization was reported previously (Tian et al., in press). Using 3AESA-linked sensor chips, dose-dependent SPR responses were detected for TRX-m1, TPPII, SHMT4, LOX2, and ACX4, and binding to the 3AESA-linked chip was competed with increasing concentrations of SA (Figures 1A–E). These five proteins also bound and crosslinked to 4AzSA, and, for all but ACX4, this binding was suppressed by increasing amounts of SA (Figures 2A–E). The demonstration that SA competes with 3AESA and 4AzSA for binding by TRX-m1, TPPII, SHM4, and LOX2 argues that these proteins exhibit authentic SA-binding activity, even though SA binding by LOX2 was relatively weak. Based on our criterion that a protein must exhibit SA-binding activity in at least two independent, different assays to be considered a true SABP, the remaining six cSABPs analyzed in this study, including ACX4, aldolase superfamily protein (At4g26530.1), glutamine synthetase 2, patellin 1, photosystem II subunit P-1, and thioredoxin superfamily protein BAS1, do not qualify.

TRX-m1 is a member of a large family of thiol:disulfide oxidoreductases; these proteins facilitate the oxidation, reduction, and/or isomerization of disulfide bonds in target proteins. This protein family includes eight cytosolic thioredoxins (designated h-type) and three types of chloroplastic thioredoxins, including one x-type, two f -type, and four m-type. Interestingly, Tada et al. (2008) identified TRX-h3 and TRX-h5 in a screen for NPR1-interacting partners and demonstrated their participation in SA-induced conversion of disulfide-linked NPR1 oligomers to NPR1 monomers.

TPPII is a serine protease belonging to the subtilisin superfamily (Book et al., 2005). This exopeptidase breaks down fragments of proteins generated by the ubiquitin—26S proteasome system (Tomkinson, 1999). The two purported SA receptors NRP3 and NPR4 have been shown to regulate the level of NPR1 via the ubiquitin—26S proteasome system (Fu et al., 2012). In animals, TPPII, together with endopeptidases like thimet oligopeptidase (TOP), appears to be essential for amino acid recycling (Tomkinson, 1999; Saric et al., 2004). Notably, Moreau et al. (2013) showed that TOP1 of Arabidopsis binds SA, resulting in suppression of its peptidase activity. Genetically altering *TOP1* expression was found to affect immunity. Other proteases also have been shown to participate in plant immune responses (van der Hoorn, 2008). Whether TPPII participates in immunity and whether SA binding modulates its function in this or other physiological processes is unknown.

SHMs, together with several other enzymes including glutamine synthetase (see below), are important components of photorespiration, which is initiated when ribulose-1,5-bisphosphate carboxylase/oxygenase (RBC) catalyzes oxygenation rather than carboxylation of ribulose 1,5 bisphosphate to generate 3-phosphoglycerate and 2-phosphoglycolate. 2-phosphoglycolate is recycled (and its products returned to the Calvin cycle) through a series of reactions, which include conversion of glycine to serine in the mitochondria by glycine decarboxylase and SHM, concomitant with the production of ammonia and CO_2_. Since re-assimilation of ammonia by the glutamine synthetase/glutamate synthase system and CO_2_ by RBC consumes both ATP and reducing power, photorespiration reduces photosynthetic efficiency (Zhu et al., 2008). However, this process plays an important role in suppressing the production of reactive oxygen species (ROS), which would otherwise be generated by the excess light energy captured in chloroplasts (Kozaki and Takeba, 1996). Highly elevated levels of ROS cause photoinhibition and cellular damage, whereas at lower levels they act as defense signals, facilitating programmed cell death during the hypersensitive response and strengthening the cell wall, which provides a physical barrier to pathogen ingress (Mittler et al., 2004; Gechev et al., 2006; O'Brien et al., 2012). Perhaps SA binding to SHM4 and/or glutamine synthetase R2 (GSR2; see below) alters their enzymatic activity and thereby helps to modulate ROS levels.

Lipoxygenases catalyze the oxygenation of polyunsaturated fatty acids. This is the first step in the biosynthesis of oxylipins, a large group of biologically active fatty acid metabolites that includes jasmonates. The first step in the synthesis of jasmonic acid (JA) is the LOX2-mediated oxygenation of linolenic acid (Bannenberg et al., 2009). Interestingly, the enzyme responsible for catalyzing the next step, allene oxide synthase (AOS), is competitively inhibited by SA (Pan et al., 1998). Perhaps SA targets both of these JA biosynthetic enzymes as part of the well-established antagonism between these two critical defense signaling hormones (Pieterse et al., 2009; Vlot et al., 2009; Robert-Seilaniantz et al., 2011).

### Identification of cSABPs using protein microarrays

**Figure 3:**
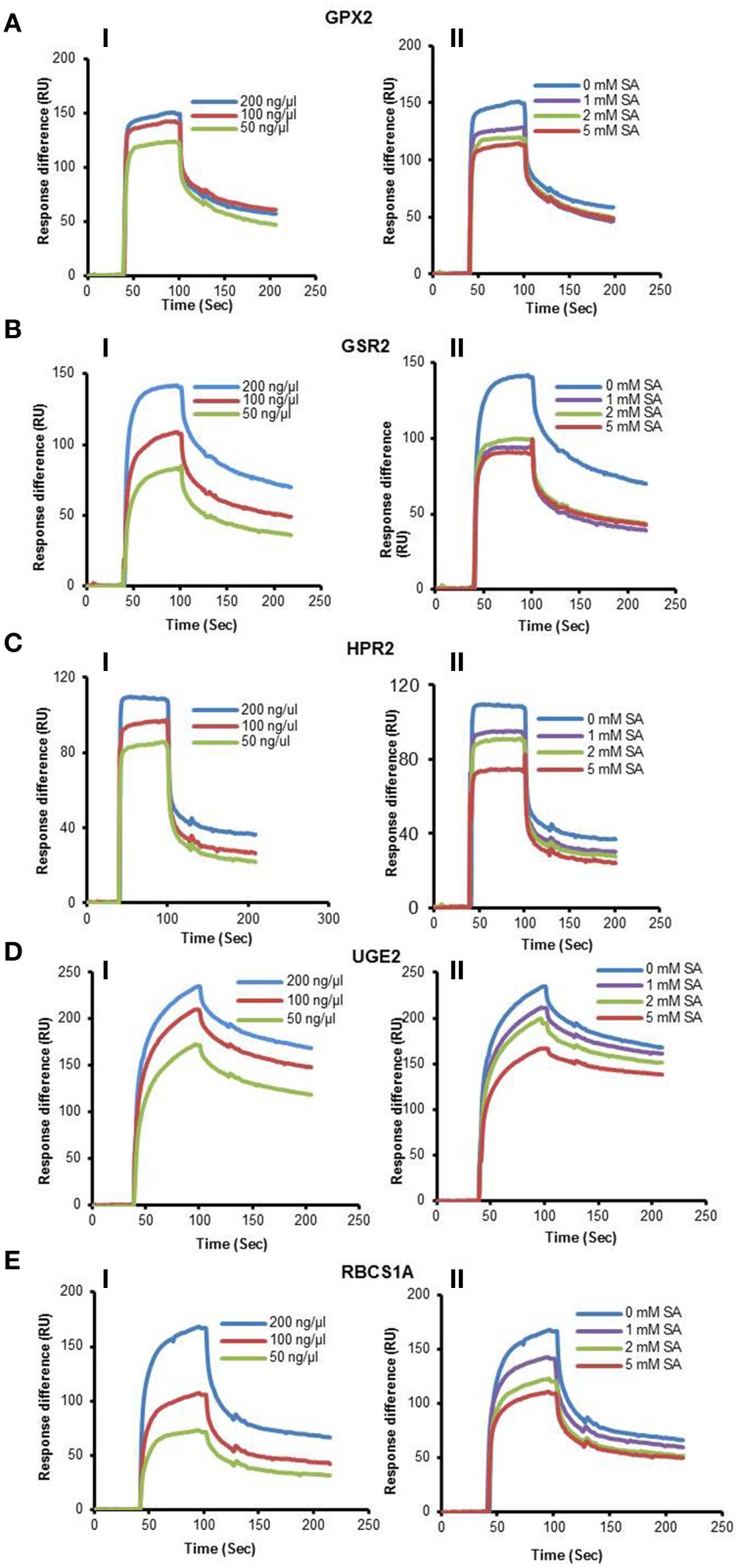
**SPR analyses of cSABPs identified by the PMA screen**. (i) Sensorgrams obtained with three concentrations (50, 100 or 200 ng/μl) of each recombinant, purified cSABP using a 3AESA-immobilized sensor chip for **(A)** GPX2, **(B)** GSR2, **(C)** HPR2, **(D)** UGE2, and **(E)** RBCS1A. (ii) Sensorgrams for each cSABP (200 ng/μl) in the absence (0 mM) or presence of three concentrations of SA (1, 2 or 5 mM) using a 3AESA-immobilized chip. The signals detected from a mock-coupled control chip were subtracted.

**Figure 4:**
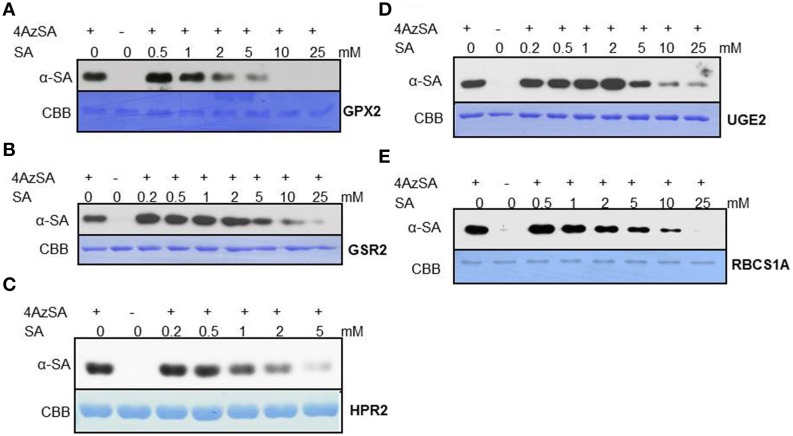
**Immuno-blot analyses monitoring SA competition of 4AzSA crosslinking to recombinant cSABPs identified by the PMA screen**. Photo-activated crosslinking of 50 ng/μl of recombinant, purified **(A)** GPX2, **(B)** GSR2, **(C)** HPR2, **(D)** UGE2, or **(E)** RBCS1A to 4AzSA (50 μM) in the absence or presence of increasing amounts of SA was detected by immuno-blotting using an α-SA antibody. Reactions without 4AzSA served as controls. Proteins stained with Coomassie brilliant blue (CBB) served as the loading control.

We also have developed a second screen for identifying cSABPs that utilizes PMAs. To reduce non-specific interactions with the test reagents, the PMAs were treated with blocking buffer before incubation with buffer lacking (the control) or containing 4AzSA, followed by UV-induced crosslinking of 4AzSA to the bound proteins. The 4AzSA-crosslinked proteins were then detected using an α-SA antibody. This strategy was used previously to screen a PMA containing 5000 Arabidopsis proteins, from which TOP1 was identified as an SABP (Moreau et al., 2013). To further enhance the detection of proteins crosslinked to 4AzSA, the incubation/reaction and washing conditions were optimized and new PMAs, containing 10,000 additional Arabidopsis proteins printed in duplicate, were screened. The results from five replicate arrays reacted with 4AzSA and five control arrays not treated with 4AzSA were used for downstream analysis. The correlation coefficient among the replicates was high (Table 2), indicating high reproducibility of the arrays. Using a cutoff of FDR < 0.01 and signal/control ratio of >1.5, 77 cSABPs were identified (Table 3). Twenty-seven cSABPs with FDR values ranging from 0.0018 to 0.0098 were selected for further characterization. Eight were successfully expressed in *E. coli* and purified by affinity chromatography and size fractionation. Five of these eight, including glutathione peroxidase 2 (GPX2), GSR2, hydroxypyruvate reductase 2 (HPR2), UDP-glucose 4-epimerase 2 (UGE2), and RBC small subunit 1A (RBCS1A), bound 3AESA and crosslinked to 4AzSA in an SA-inhibitable manner, indicating that they are SABPs (Figures 3, 4). Analysis of protein phosphatase 2A (PP2A), GS2, and an α/β hydrolase superfamily member failed to meet the criteria for designation as SABPs (data not shown).

**Table 2:** **Correlation of protein microarrays**.

**Microarray**	**+4AzSA#1**	**+4AzSA#2**	**+4AzsA#3**	**+4AzSA#4**	**+4AzsA#5**	**−4AzsA#1**	**−4AzsA#2**	**−4AzsA#3**	**−4AzsA#4**	**−4AzsA#5**
+4AzSA#1	1	1	1	1	0.9	0.9	1	0.9	0.9	0.9
+4AzSA#2	1	1	1	1	0.9	0.9	1	1	0.9	0.9
+4AzSA#3	1	1	1	1	0.9	0.9	0.9	0.9	0.9	0.9
+4AzSA#4	1	1	1	1	0.9	0.9	1	0.9	0.9	0.9
+4AzSA#5	0.9	0.9	0.9	0.9	1	0.8	0.9	0.9	0.9	0.9
−4AzSA#1	0.9	0.9	0.9	0.9	0.8	1	0.9	0.9	0.9	0.9
−4AzSA#2	1	1	0.9	1	0.9	0.9	1	1	1	1
−4AzSA#3	0.9	1	0.9	0.9	0.9	0.9	1	1	1	1
−4AzSA#4	0.9	0.9	0.9	0.9	0.9	0.9	1	1	1	1
−4AzSA#5	0.9	0.9	0.9	0.9	0.9	0.9	1	1	1	1

Pearson correlation coefficient were calculated among the 10 protein microarrays. Five replicated microarrays with 4AzSA:+4AzSA#1, +4AzSA#2, +4AzSA#3, +4AzSA#4 and +4AzsA#5; five replicated microarrays without 4AzSA: −4AzSA#1, −4AzSA#2, −4AzSA#3, −4AZSA#4, and −4AzSA#5.

**Table 3:**
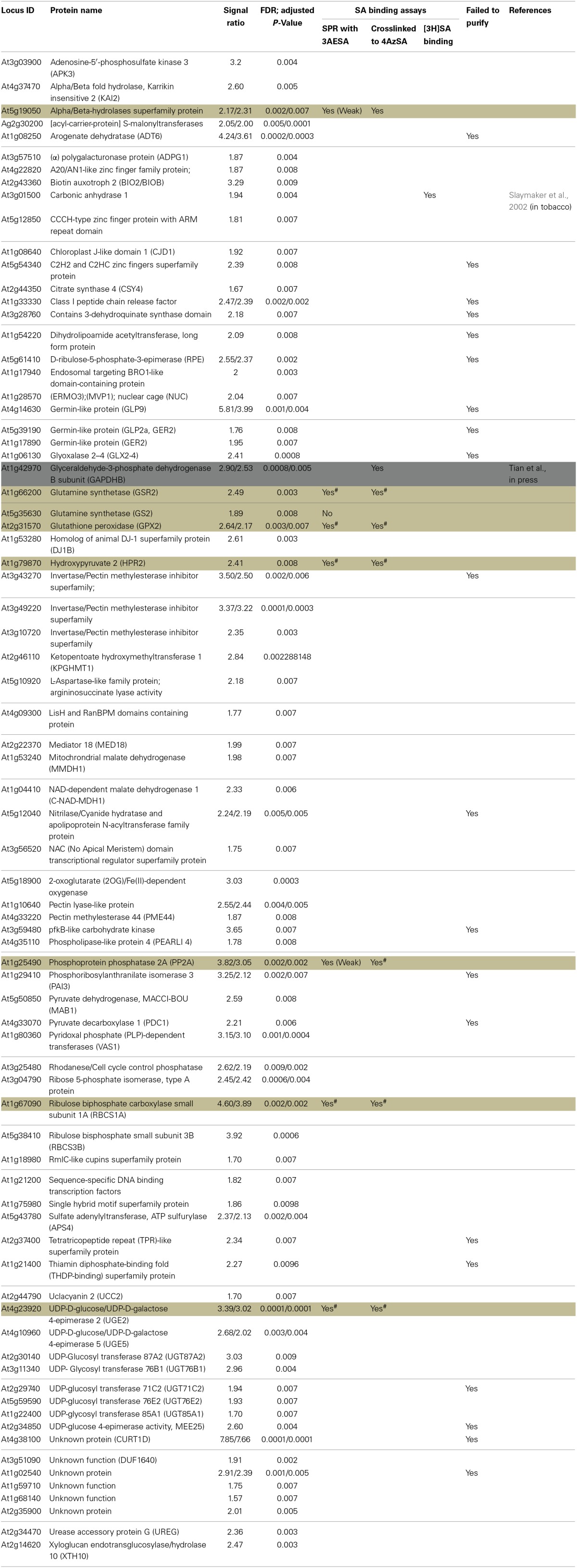
**List of cSABPs identified using protein microarrays**.

FDR, false discovery rate.

Signal ratio corresponds to the ratio of fluorescent signal obtained on PMA incubated with 4AzSA divided by the signal obtained on PMA incubated without 4AzSA.

^#^Competable with SA.

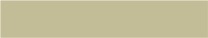
Characterized in this study.

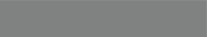
Previously reported cSABPs/SABPs.

GPXs, like the SAPBs catalase and ascorbate peroxidase (Chen et al., 1993; Durner and Klessig, 1995), help regulate cellular redox by scavenging H_2_O_2_. In Arabidopsis, GPX2 is located in the cytoplasm and its expression is induced by abiotic stresses, including salt, osmotic stress, and heavy metals. It also is induced by SA, but not by other hormones such as JA, abscisic acid or indole acetic acid (Milla et al., 2003). Whether SA inhibits GPX2 activity like it does for the other two H_2_O_2_-scavenging enzymes is not known.

Glutamine synthetases play key roles in nitrogen metabolism, including the assimilation of inorganic nitrogen via conversion of ammonia into glutamine. Two glutamine synthetases were identified in the PMA screen: GSR2 and GS2 (Table 3). GS2 was also identified by immuno-selection of 4AzSA-crosslinked proteins (Table 1). However, while GS2 failed to exhibit binding to 3AESA in the SPR assay, GSR2 was found to be an SABP (Figures 3B, 4B). GS2 is located in chloroplasts, where it plays an important role in the reassimilation of ammonia released during photorespiration (Wallgrove et al., 1987). Whether GSR2 also plays a role during photorespiration is unknown.

HPR2 is another enzyme involved in photorespiration. It, like the peroxisome-localized HPR1, converts hydroxypruvate to glycerate, which, upon phosphorylation, is returned to the Calvin cycle as an intermediate. HPR2 is a cytosolic enzyme (Timm et al., 2008).

UGEs interconvert UDP-glucose and UDP-galactose. Arabidopsis contains five UGE isoforms, which are divided into two clades; one clade contains UGE1 and UGE3, and the other contains UGE2, UGE4, and UGE5. UGE 2 and UGE5 were both identified in the PMA screen (Table 3), and further analyses confirmed that UGE2 is an SABP (Figures 3D, 4D). UGE2 and UGE4 are reported to cooperate in providing UDP-galactose for cell wall biosynthesis and growth, while UGE5 may play a role during abiotic stress (Rosti et al., 2007).

Several members of the RBC small subunit (RBCS) family were identified in the PMA screen and/or at least once in the 4AzSA/immuno-selection screens. These include RBCS1A, RBCS1B, and RBCS3B (Tables 1, 2). Initially we discounted the repeated identification of RBCS in our 4AzSA/immuno-selection screen, assuming that they were non-specifically selected because of their overwhelming abundance in soluble protein extracts. The likelihood that they were all false positives decreased significantly when RBCS1A and RBCS3B were identified repeatedly on the PMA screen, as protein abundance does not influence these results. Further characterization of RBCS1A confirmed that at least this RBCS is an SABP (Figures 3E, 4E). Since SA is synthesized in chloroplast and has been linked to several metabolic processes, including redox homeostasis and photosynthesis (Mateo et al., 2006; Janda et al., 2014), the discovery that RBC, a central enzyme in photosynthesis, binds SA is perhaps not that surprising.

### NPR1 is an SABP

**Figure 5:**
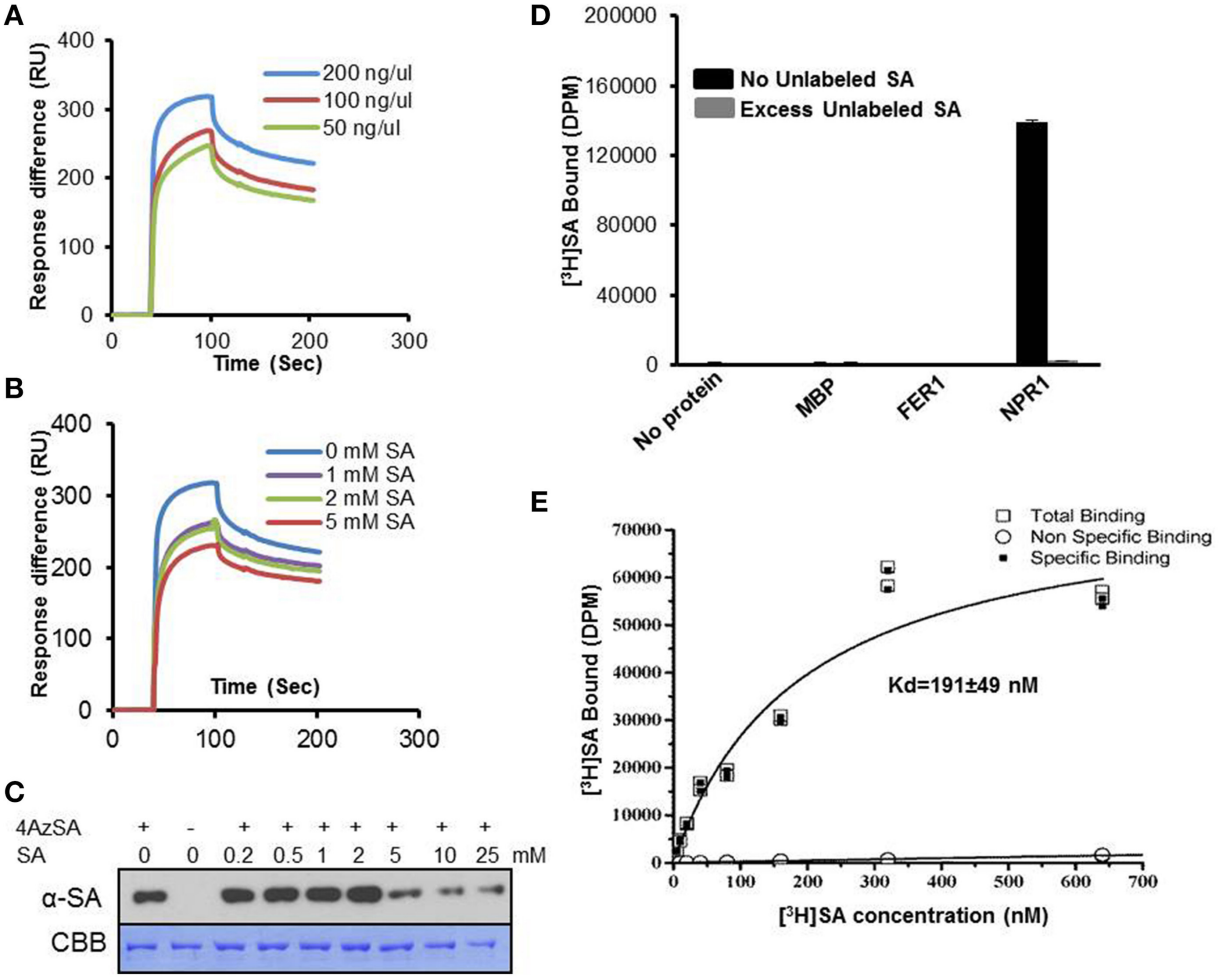
**SA-binding activity of NPR1 detected by SPR, photo-activated crosslinking to 4AzSA, and binding of [^3^H]SA. (A)** Sensorgrams obtained with three concentrations of recombinant, purified NPR1 (50, 100 or 200 ng/μl) using a 3AESA-immobilized sensor chip. **(B)** Sensograms for NPR1 (200 ng/μl) in the absence (0 mM) or presence of three concentrations of SA (1, 2 or 5 mM) on a 3AESA-immobilized chip. The signals detected from a mock-coupled control chip were subtracted. **(C)** Photo-activated crosslinking of 50 ng/μl of NPR1 to 4AzSA (50 μM) in the absence or presence of increasing amounts of SA was detected by immuno-blotting using an α-SA antibody. Reactions without 4AzSA served as controls. Proteins stained with Coomassie brilliant blue (CBB) served as the loading control. **(D)** Binding of [^3^H]SA (200 nM) by 0.20 μg/μl His_6_-MBP-tagged NPR1 in the absence or presence of a 10,000-fold excess of unlabeled SA was determined by size-exclusion chromatography. Chromatography reactions with [^3^H]SA with no protein, with MBP, or with His_6_-MBP-tagged ferretin 1 (FER1), which does not bind SA, served as negative controls. Error bars represent SE values calculated from three replications of a single experiment. The [^3^H]SA binding assay with NPR1 was repeated at least four times with similar results. **(E)** Dissociation constant (Kd) of 0.125 μg/μl NPR1 binding to SA was determined by size-exclusion chromatography with different concentrations of [^3^H]SA. No protein with different concentrations of [^3^H]SA was used as non-specific binding control. Two replicates in a single experiment were used to calculate Kd; the experiment was done twice.

Given the conflicting reports on whether NPR1 binds SA (Fu et al., 2012; Wu et al., 2012), we revisited this matter using the methods we have optimized/developed for identifying SABPs. Recombinant *Arabidopsis* NPR1 exhibited a dose-dependent SPR response on 3AESA-linked sensor chips, and NPR1 binding to 3AESA was competed by increasing concentrations of SA (Figures 5A,B). In addition, NPR1 bound and was crosslinked to 4AzSA; this crosslinking was suppressed by increasing levels of SA (Figure 5C). NPR1's SA binding ability was further confirmed using a classical method for identifying low molecular weight ligand binding proteins, namely size exclusion chromatography. NPR1 bound [^3^H]SA, thereby excluding this ligand from entering the interior of the matrix bead. Furthermore, excess unlabeled SA competed with [^3^H]SA for binding to NPR1 (Figure 5D). It binds SA with relatively high affinity with an apparent Kd on 191 ± 49 nM (Figure 5E). Therefore, based on these three independent assays, we conclude that NPR1 is an SABP.

## Discussion

In this study, we report the identification of nine new SABPs, as well as the generation of a large pool of cSABPs, many of whose SA-binding properties have yet to be tested. Most of these proteins were identified through recently developed high-throughput screens that utilize photo-activated crosslinking to stabilize the interaction between cellular proteins and 4AzSA. Biochemical and biological tests have previously demonstrated that 4AzSA mimics SA, as it induces expression of the prototypic SA-responsive *PR-1* gene and competes with [^3^H]SA for binding to a known SABP, AtMES9 (Tian et al., 2012). For all nine SABPs, their ability to crosslink to 4AzSA in the initial screens was subsequently shown to represent authentic SA binding since (i) this crosslinking was suppressed in the presence of SA, and (ii) these proteins exhibited SA-inhibitable binding to 3AESA, which was covalently linked to an SPR sensor chip through an amide bond.

SPR is a highly sensitive method for identifying interactions that are weak and/or transient, quantitatively measuring interactions in real time. Photo-affinity labeling with 4AzSA also is well suited for identification of interactions that are weak and/or transient since 4AzSA binding is captured by photo-activated crosslinking. However, a common problem with photo-affinity labeling is non-specific labeling, which can lead to many false positives (Kotzyba-Hilbert et al., 1995). Non-specificity and the resulting high number of false positives are a general problem with high-throughput screens, including those employing PMAs. This problem was particularly severe in our initial screen for cSABPs using SA linked to a PharmaLink matrix. Despite our attempts to remove proteins non-specifically bound to the matrix via excessive washing of the SA-linked matrix with 4-hydroxy benzoic acid, a biologically inactive SA analog, a large portion of the proteins subsequently eluted with SA were found to be false positives upon further characterization. This setback prompted us to develop new screens that rely upon stabilizing the interaction between cellular proteins and 4AzSA through photo-activated crosslinking. Of the 35 cSABPs identified in the 4AzSA/immuno-selection screen, 19 were further analyzed to varying degrees in this and previous reports (Table 1; Tian et al., 2012, in press). Eleven of these 19 proteins met the criteria for designation as a true SABP, as they exhibited SA binding in at least two different assays. The nearly 60% success rate for this screen is somewhat misleading, since six of the 11 were members of just two protein families - GST and GAPDH. However, of the 16 cSABPs yet to be characterized, catalase and carbonic anhydrase are highly likely to be SABPs, given that their tobacco orthologs are SABPs (Chen et al., 1993; Durner and Klessig, 1995; Slaymaker et al., 2002). The results from our PMA screening strategy also appear promising, as 77 cSABPs were identified in a screen of 10,000 proteins. Only a small portion of these cSABPs have been characterized further, due to the recent optimization of this screen and technical difficulties generating the recombinant proteins. However, of the eight cSABPs analyzed thus far, five met the criteria to be designated as SABPs. Together, these findings suggest that both the 4AzSA/immuno-selection and the PMA screening strategies will yield very workable numbers of candidates that have a significant probability of being SABPs.

It is interesting to note that four of the nine newly identified SABPs are associated with redox regulation. The interplay between SA and redox homeostasis was first revealed with the discovery that SA inhibits the activity of two major H_2_O_2_-scavenging enzymes in tobacco: catalase, which is the first SABP identified (Chen et al., 1993), and ascorbate peroxidase (Durner and Klessig, 1995). Further linking SA and redox status was the discovery by Dong and coworkers that translocation of NPR1 from the cytoplasm to the nucleus, which is required for NPR1 to play its central positive role in SA-mediated immunity, is redox regulated (Mou et al., 2003). Many subsequent studies have documented the interplay among SA, ROS, redox homeostasis, and the activation of immune responses (Mateo et al., 2006; Dat et al., 2007; Vlot et al., 2009; Xu and Brosche, 2014). Of the four redox-associated SABPs, GPX2 is an H_2_O_2_ scavenger, while TRX-m1 is an oxidoreductase that regulates disulfide bond formation/deformation in target proteins. SHM4 and GSR2 function in photorespiration, which plays a critical role in preventing cellular damage due to overproduction of ROS generated by excess light energy. Over production of ROS also causes photoinhibition due to damage to the photosynthetic apparatus, particularly to photosystem II. Notably RBCS1A of the key photosynthesis enzyme RBC was found to bind SA.

Unlike animal systems, relatively few plant hormones have been identified with each mediating multiple biochemical and physiological responses. Our understanding of the biochemical and molecular mechanisms of phytohormone perception and signaling also is relatively rudimentary in comparison to what is known in animals. While receptors for SA have recently been reported (Fu et al., 2012; Wu et al., 2012), the results from our studies suggest that many of SA's effects are mediated though a large number of SABPs whose biochemical/enzymatic activities are altered by SA binding. Classical receptors have been discovered for most phytohormones over the past several decades. However, we suspect that some of these phytohormones, like SA, utilize additional protein targets either in conjunction with or instead of their known receptors to mediate some of their myriad effects. Since the approaches and methods developed/optimized for the identification of SABPs are applicable to the identification of proteins that bind other low molecular weight compounds/ligands, such as other plant (or animal) hormones, their future use should clarify whether the SA signaling network serves as a paradigm for other phytohormones. In fact, we have used these approaches/methods to identify several novel human targets of the most used drug worldwide, namely aspirin, which is rapidly metabolized to SA. Natural derivatives of SA are found in several medicinal plants, which are used extensively in traditional medicine.

## Author contributions

Daniel F. Klessig, Miaoying Tian, Murli Manohar, Sang W. Park, and Magali Moreau designed the research; Murli Manohar, Miaoying Tian, Magali Moreau, Sang W. Park, Hyong W. Choi, Muhammed Asif, Patricia Manosalva, Caroline C. von Dahl, Kai Shi, and Inish O'Doherty performed SA-binding analyses; Giulia Friso and Klass J. van Wijk performed the mass spectroscopy analyses; Shisong M and Savithramma P. Dinesh-Kumar provided the protein microarrays; Murli Manohar, Miaoying Tian, Magali Moreau, Sang W. Park, Giulia Friso, Zhangjun Fei, Hyong W. Choi, Frank C. Schroeder, and Daniel F. Klessig analyzed the data; and Daniel F. Klessig, Murli Manohar, Miaoying Tian, and Magali Moreau wrote the paper.

### Conflict of interest statement

The authors declare that the research was conducted in the absence of any commercial or financial relationships that could be construed as a potential conflict of interest.
